# Determinants of mTOR inhibitor therapy in AIDS-associated malignancies

**DOI:** 10.1186/1750-9378-7-S1-O9

**Published:** 2012-04-19

**Authors:** Dirk P Dittmer, Blossom Damania, Sang-Hoon Sin, Debasmita Roy

**Affiliations:** 1 Department of Microbiology and Immunology, Lineberger Comprehensive Cancer Center, Center for AIDS Research, University of North Carolina at Chapel Hill, Chapel Hill, NC, USA

## 

Rapamycin/Sirolimus™ leads to the regression of transplant-associated Kaposi sarcoma (KS). It also leads to disease stabilization in HIV-associated KS. Case reports and a wealth of preclinical studies support rapamycin’s efficacy also in AIDS associated lymphoma, such as primary effusion lymphoma (PEL). Rapamycin inhibits the mammalian target of rapamycin (mTOR) and papamycin derivatives are approved for the treatment of mantle cell lymphoma and other cancers. It is not universally effective against all solid tumors. Even within this group of clinically responsive cancers, there are exceptions of cases or cell models in which this drug or its derivatives (rapalogs) fail.

We hypothesized that genetic alterations of the tumor will determine the response to rapalogs. We used a novel KS TMA (from the AIDS cancer specimen resource (ACSR) to evaluate the PI3K/Akt/mTOR pathway in KS and identified a unique pattern of protein activation that is associated with sustained expression and phosphorylation of the PTEN tumor suppressor protein. We used Affymetrix array-based comparative genome hybridization (CGH) and targeted sequencing to query genomic loci of members of the mTOR pathway, which confirmed the absence of genetic alterations, which in other cancers have been associated with mTOR pathway activation. We conclude that in KS, PEL and perhaps other virus-associated cancer the mTOR pathway is activated post-translationally, which is not as permanent and does not have the same impact as for instance a deletion in PTEN. This may explain the unique sensitivity of these tumor types to rapalogs.

We established a novel animal model of KS (L1T2 cells), which develops tumors with short latency and in which the KS associated herpesvirus (KSHV) is maintained in each tumor cell. We evaluated multiple rapalogs (Sirolimus™, Temsirolimus™, Everolimus™), Tacrolimus/FK506 and Doxil™ in this model. We found sustained, but reversible tumor suppression by the rapalogs, which was associated (a) with inhibition of mTOR activity and (b) with reduced neo-angiogensis. In this model rapalogs outperformed Doxil™, presumably because of a combined effect on endothelial lineage KS tumor cells and endothelia lineage tumor vasculature. This represents the first animal test of rapalogs in a human KS tumor model, it validates novel biomarkers and provides a strong rational for further clinical development and testing of rapalogs in AIDS-associated Kaposi sarcoma.

As rapamycin has recently become affordable and is available in developing countries, where the majority of KS-associated mortality occurs, it is one of the few new anti-cancer drugs with a cost/benefit profile that is affordable for global cancer treatment.

